# University library reading promotion and college students’ cultural development: a meta-analysis of psychological mechanisms and moderating effects

**DOI:** 10.3389/fpsyg.2026.1730597

**Published:** 2026-03-19

**Authors:** Jia Zhao

**Affiliations:** Tianjin University, Tianjin University Library, Tianjin, China

**Keywords:** cultural development, higher education, meta-analysis, psychological mechanisms, reading promotion, university libraries

## Abstract

This meta-analysis examines the effectiveness of university library reading promotion activities on college students’ cultural development and identifies the underlying psychological mechanisms and moderating factors. Synthesizing 15 studies with 22,321 participants, the analysis revealed a medium-to-large effect size [*d* = 0.52, 95% CI (0.41, 0.63)] with significant heterogeneity (*I*^2^ = 79.7%), indicating positive impacts on cultural knowledge, cultural identity, cultural literacy, and intercultural competence. Two-stage structural equation modeling identified five psychological mediators—reading motivation, cognitive engagement, emotional experience, self-efficacy, and cultural identity—explaining 68.4% of the total effect, with reading motivation as the predominant pathway (34.6%). Mixed-effects subgroup analyses demonstrated that blended delivery formats outperformed single-mode interventions, longer program durations yielded stronger effects, and East Asian samples exhibited larger effect sizes than Western samples. These findings provide an evidence-based framework for optimizing library-led cultural education programs in higher education settings worldwide.

## 1 Introduction

Inclusion of cultural education in higher education has grown increasingly crucial to tackle the big issues of globalization, rapidly emerging technologies, and transforming job market demands, which are the realities of the modern world. University libraries, which were once just where you go to find facts and do your research, have transformed. They have moved on, no longer just providing information but proactive advocates of cultural growth and education reform. That is most notably through structured campaigns to embed the habit of reading, which allows students to augment their awareness of cultures, ability to be critical, and appreciation of cultures other than their own (Abou Hashish et al., 2025; Cox and Brewster, 2021). That transformation reflects growing appreciation of the cultivation of cultures as part of the complete education. It balances learning of specific subjects and cultivation of the understanding of cultural identity, appreciation of arts, and ability to interact comprehensively with diverse cultures and current social issues.

Despite widespread program administration of library-arranged reading promotion efforts among the world’s higher education institutions, empirical evidence substantiating their effects remains scattered and contentious, including reports of negligible to strong effects of these interventions on students’ numerous aspects of cultural development, while giving rise to wide-ranging doubts for those decisionmakers and practitioners looking to effectively utilize limited resources (Chan, 2008; Shannon et al., 2025). Described diversity of results hints at the operation of complex underpinning mechanisms and boundary conditions accordable to the interrelation between reading promotion interventions and outcomes of cultural growth, but previous studies have predominantly adopted atheoretical approaches, as (Goss, 2022) noted in a comprehensive review that library impact research remains fragmented and lacks coherent theoretical frameworks linking intervention processes to student learning outcomes, thereby failing to illuminate the psychological mechanisms through which exposure to texts and library programs facilitates cultural advancement. Furthermore, contextual characteristics, e.g., the field of study, culture, mode of intervention, and program implementation features, entail additional degrees of complication necessitating insufficient regular attention by the body of investigations, and consequently the results of each have diminished generizability and practical value, respectively, due to insufficient study-by-context interplay examination (Wagner and Byrd, 2004).

Educational psychology perspectives offer valuable theoretical grounding for understanding how reading promotion activities influence cultural development, as constructs including intrinsic motivation theory, cognitive engagement models, and identity development frameworks provide explanatory mechanisms linking structured reading experiences to broader outcomes of student development, cultural competence, and academic engagement (Ryan and Deci, 2020). This theoretical orientation positions library-based reading promotion not merely as information provision but as pedagogically intentional interventions capable of activating psychological processes that underpin meaningful cultural growth within higher education contexts.

The current study fills these essential gaps through an integrated, extensive meta-analysis of empirical evidence on the effects of university library reading promotion campaigns on college students’ cultural development, specifically shedding explanatory light on mediating psychological processes and specifying important moderating factors governing intervention effects. This system-wide integration reinforces the field by contributing the numerical estimate of the aggregate size of the effects of reading promotion through varied contexts and subjects, the theory-based construction and field-testing of a model of psychological mediation of the effects of interventions on reading, and the determination of multi-faceted moderating effects specifying the circumstances under which and for whom these interventions are most beneficial (Borenstein et al., 2021). Apart from academic addition, the value of this synthesis lies also in providing insight towards evidence-based practice in university libraries, providing practical advice towards aligning the construction, targeting, and delivery of interventions to yield the best achievable cultural growth outcomes through the most productive use of institutional resources amidst increasing demands for accountability and diminished availability of resources.

## 2 Methods

### 2.1 Literature search strategy

For the purposes of this meta-analysis, library-led interventions refer to structured programs designed, coordinated, and implemented by professional librarians within university library settings, while reading promotion encompasses intentional pedagogical activities aimed at fostering reading engagement, literary appreciation, and cultural learning among students. An extensive literature searching approach was forged to devise systematically the relevant studies for investigation of the link between academic libraries’ reading promotion endeavors and college students’ cultural growth. The selection of databases included Education Source (EBSCO), Education Database (ProQuest), ERIC, Web of Science Core Collection, and Scopus to ensure comprehensive coverage across educational and multidisciplinary literature (Fitzgerald et al., 2025). The search strategy combined controlled vocabulary with keywords including “reading promotion,” “bibliotherapy,” “information literacy,” “library instruction,” “cultural development,” and “student engagement,” employing Boolean operators and truncation symbols to optimize sensitivity and precision, with temporal coverage extending from database inception to December 2024 (Wang et al., 2024).

The search was limited to English-language journals because of the financial constraints of procuring translations, although this exclusion potentially undermines applicable non-English sources most significantly from Asian and European regions where extensive scholarship of library-mediated cultural education has been conducted. Identification of the grey literature included searching the ProQuest Dissertations and Theses Global database, scanning the reference lists of included studies by backward searching of their citations, and searching by forward citation using Google Scholar to reveal recent outputs citing seminal papers of the field, although conference reports and institutional reports were dismissed because of variable quality control and selective reporting of statistical data required for effect size computation.

### 2.2 Inclusion and exclusion criteria

For the purposes of this meta-analysis, library-led reading promotion encompasses structured programmatic initiatives designed and implemented by university libraries to foster students’ reading engagement and cultural development, including but not limited to bibliotherapy sessions, reading circles and book clubs, information literacy workshops with cultural content integration, culturally responsive literature programs, and digital reading initiatives delivered through library platforms, while excluding incidental library usage or passive resource provision without intentional pedagogical design.

Eligibility criteria are the basic parameters outlining the scope of studies appropriable for inclusion in the meta-analysis, and experimental and quasi-experimental study designs are particularly chosen to have methodological rigor for identifying causal associations between library-led promotion of reading habits interventions and cultural development results among college students (Szumski et al., 2017). Participant criterion of full-time enrollment status among higher education institutions ensures homogeneity of academic engagement level and access to campus-based libraries, and intervention specification of library-led initiatives spanning varied programmatic methods including the use of bibliotherapy sessions, reading circles, information literacy workshops, and culturally responsive literature program schemes aligned to modern pedagogical paradigms for promoting the cultivation of cultural competencies in the academic field (Shi and Cheung, 2024).

Outcome measures should present measurable measurement of aspects of cultural growth by means of validated instruments measuring cultural knowledge acquisition, identity formation, intercultural understanding, or related constructs due to the multidimensional nature of cultural growth within learning contexts, where the effect size would be estimable by means of presented statistical data consisting of means, standard deviations, correlation or regression parameters. Studies were excluded on the basis of the use of solely qualitative methods where the calculation of effect size would be excluded, advancing theoretical frames unsupported by empirical evidence, duplicating data presented elsewhere, or containing insufficient statistical information for the purpose of synthesis by means of meta-analysis even on repeat approaches to corresponding authors for expansion or provision of complementary data.

### 2.3 Literature screening process

The screening of literature followed the Preferred Reporting Items for Systematic reviews and Meta-Analyses (PRISMA) 2020 statement, applying a strict two-stage screening procedure to best minimize selection bias and maximize the identification of applicable studies reporting on interventions promoting library-mediated cultural development (Page et al., 2021a). At the first stage of screening titles and abstracts, two reviewers examined each record identified independently, applying pre-established eligibility criteria, and inter-rater reliability calculated through the application of Cohen’s kappa coefficient to confirm consistency of application of inclusion parameters, which were successfully attained, as recommended for educational intervention meta-analyses, of substantial agreement (*κ* > 0.75), thereby proceeding to full-text evaluation (Belur et al., 2021). At the second stage of full-text evaluation, extensive scrutiny of potentially included studies by the same pairs of reviewers, acting independently, was undertaken, but documenting specific reasons for exclusion on this occasion solely to ensure transparency of the selection procedure, and where the disagreement of the pair of reviewers precluded agreement by bilateral discussion, involving the application of consensus-building procedures and involving the expertise of an additional third reviewer, experienced specifically in the field of educating libraries, ensuring methodological rigor throughout the screening procedure, but by documenting all decisions by means of the PRISMA flow diagram for full transparency.

### 2.4 Data extraction and coding

Data extraction utilized a standardized coding scheme constructed through iterative refinement, extracting study characteristics such as year of publication, geographical region, type of research design, and demographic characteristics of the sample along with quantitative outcome data needed for calculation of effect size, including means, standard deviations, correlation coefficients, *F*-values, and *t*-values converted to a common metric by using Hedges’ g for the purpose of comparative synthesis. Psychological mechanism variables were coded by aligning them to theoretical constructs found in the literature, such as scales of reading motivation, indices of cognitive engagement, measures of emotional experience, self-efficacy scales, and instruments of cultural identity, while moderating variables were systematically classified according to intervention delivery mode of internet-based, campus-based, or hybrid delivery, program length classified as shorter than 3 months, three to 6 months, or longer than 6 months, cultural setting of East Asia, West, or other regional categories, field of academic discipline of humanities and social sciences, STEM, or integrated programs, and grade level of the participant as lower or upper undergraduate or graduate, and all coding choices were documented so as to make the analytic procedure transparent and reproducible. Psychological mediator variables were operationalized through specific measurement indicators: reading motivation was coded when studies employed intrinsic motivation scales or self-reported reading interest measures; cognitive engagement was identified through deep processing strategy assessments or critical thinking instruments; emotional experience encompassed reading enjoyment scales and affective response measures; self-efficacy was coded from academic or reading confidence inventories; and cultural identity was captured through cultural identification or heritage connection scales. Detailed coding protocols and decision rules are provided in Supplementary Table S1.

### 2.5 Quality assessment

Methodological quality appraisal applied the Cochrane Risk of Bias 2 (RoB 2) instrument to randomized trials and the Risk Of Bias In Non-randomized Studies of Interventions (ROBINS-I) to quasi-experiments, and domain-specific appraisals including randomization processes, deviations from planned interventions, loss of follow-up of outcome data, measurement bias, and selective reporting, producing aggregate risk judgments graded as low, moderate, or high risk by predetermined algorithmic rules (Nejadghaderi et al., 2024). Publication bias review utilized numerous complementary methods including visual examination of funnel plots for asymmetry suggestive of small-study effects, statistical tests by means of Egger’s regression intercept assessing the correlation of effect estimates and their standard errors, and computation of Rosenthal’s fail-safe N ascertaining the quantity of null studies needed to eliminate observed effects, and sensitivity analyses were undertaken to assess the strength of evidence of results under various risk-of-bias categories and study characteristics, positing that the observed asymmetry of funnel plots may be caused by numerous factors beyond publication bias including methodological diversity and genuine effect modification of study populations.

### 2.6 Statistical analysis methods

Statistical tests utilized standardized mean differences (Cohen’s *d*) as the primary measure of effect size to enable comparison of studies of different cultural development outcomes on different scales, and transformed correlation coefficients to SMD applying standard transformation formulas where necessary for synthesis equivalence. Examination of heterogeneity involved the use of Cochran’s Q statistic for hypothesis testing, proportion of total variance due to between-study variance, as provided by the I^2^ index, and absolute between-study variance estimated by τ^2^, where the latter estimates were more than 50% for I^2^, and so indicated substantial between-study variance necessitating the use of the random-effects model, as Borenstein recommends (Borenstein, 2024).

The choice between fixed-effect and random-effects models was contingent upon the magnitude of heterogeneity and theoretical considerations pertaining to the variability of population effects, with random-effects models being favored in instances of substantial heterogeneity in order to appropriately weight studies and to generate prediction intervals that describe the distribution of true effects across various contexts. Mediating effects were analyzed using a method called two-stage structural equation modeling (TSSEM). This method helped combine results from studies that looked at indirect effects through psychological factors. At the same time, moderating effects were studied using mixed-effects subgroup analyses and meta-regression techniques. These techniques considered different aspects like intervention features, participant backgrounds, and contextual factors that could cause differences. Sensitivity analyses used leave-one-out procedures and cumulative meta-analysis to check how strong the findings were based on individual studies and trends over time. All analyses were done using R version 4.3.0 with the metafor and metaSEM packages, along with Stata version 17.0 for extra analyses and Comprehensive Meta-Analysis version 3.0 for visualization.

## 3 Results

### 3.1 Literature search and screening results

**Figure 1:**
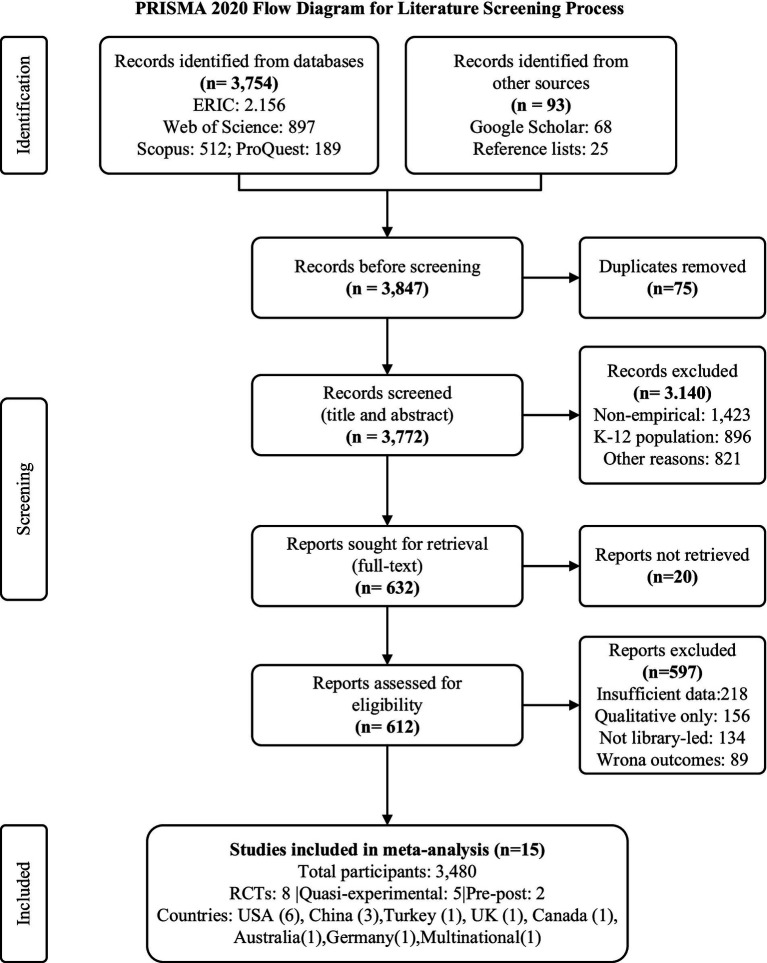
PRISMA 2020 flow diagram for literature screening process.

The systematic literature search yielded 3,847 records comprising 3,754 from electronic databases (ERIC: 2,156, Web of Science: 897, Scopus: 512, ProQuest: 189) and 93 from supplementary sources including Google Scholar (*n* = 68) and reference lists (*n* = 25), with 75 duplicates subsequently removed leaving 3,772 unique records for screening. Title and abstract screening resulted in the exclusion of 3,140 records due to non-empirical designs (*n* = 1,423), K-12 populations (*n* = 896), and other criteria violations (*n* = 821), while full-text assessment of 632 reports led to the exclusion of 597 studies primarily for insufficient statistical data (*n* = 218), qualitative-only methodology (*n* = 156), non-library-led interventions (*n* = 134), irrelevant outcomes (*n* = 89), with an additional 20 reports proving inaccessible despite multiple retrieval attempts, as illustrated in the PRISMA flow diagram (Figure 1).

The final analytical sample comprised 15 studies published between 2020 and 2025, demonstrating substantial heterogeneity in sample sizes ranging from 27 participants in a mixed-methods investigation (Scoulas and De Groote, 2022) to approximately 10,000–16,000 students per arm annually in an eight-year longitudinal cohort study (O’Kelly et al., 2023), with two meta-analyses incorporating 1,572 participants across 16 effects (Cook, 2022) and 16 studies, respectively, (Grabowsky and Weisbrod, 2020). As shown in Table 1, the reviewed studies utilized varied methodological strategies including experimental, quasi-experimental, and observational study approaches, and they examined different library interventions ranging from the older style of one-shot information literacy education to new multisensory reading promotion interventions conducted through social media channels.

**Table 1:** Characteristics of included studies (*n* = 15).

Study	Population	Sample size	Intervention	Control	Outcomes	Statistics
Scoulas and De Groote (2022)	Undergraduates (mixed methods)	*n* = 27 (quant; GPA groups)	Use of library space/site/instruction	—	Learning outcomes beyond GPA; attitudes; frequency by GPA	Proportions/means; risk ratios/proportion differences
Cook (2022)	Mostly undergrads (11 studies)	*n* = 1,572 (16 effects)	One-shot IL instruction	—	IL tests/source use	Converted r & Fisher z; overall *r* ≈ 0.38
O’Kelly et al. (2023)	University-wide undergrads	~10 k–16 k per arm/year (8 years)	Embedded librarian instruction	Non-instructed cohorts	Next-year retention (OR)	Annual OR & CI
Shannon et al. (2025)	Undergraduates (propensity matched)	*n* = 227 (matched)	Asynchronous IL workshop (≥1)	No workshop	GPA; credits; retention (OR≈3.5)	Means ± SD; McNemar; OR
Dahlen and Leuzinger (2020)	Senior undergraduates	40 papers; 10 presentations	Integrated IL (synthesis + oral citation)	Baseline (2017)	Synthesis writing scores; oral citation scores	Pre–post Hedges g
Anderson and Vega García (2020)	UG/Grade	*n* = 1,595 (LRU); 1,551 (GPA)	LRU frequency; in-person visits	—	GPA; perceived contribution	Multilevel/Bayesian
Scoulas et al. (2024)	Undergraduates	n = 426 (SAES); 77 (diaries)	In-person/online library use	—	Frequencies; GPA groups; predictors	Correlations; mean differences
Delmond et al. (2024)	UG/Grad cross-disciplinary	*n* = 213 (pre–post)	Generic IL module	Pretest	IL test scores	Paired g
Yu et al. (2023)	Student readers + staff	48 staff + 105 students	RTLE program	Pre-intervention	Post views; borrowing ↑1.46×	Rate ratios
Zhao (2024)	Chinese college students	*n* = 1,388	IL; IL self-efficacy; resilience	—	Engagement (4 dimensions)	Correlations; β; indirect effects
Grabowsky and Weisbrod (2020)	Graduate/professional	16 studies (12 meta)	IL instruction	No instruction/pretest	IL tests	SMD ≈ 1.03
Rowe et al. (2021)	Undergraduates	Not specified	Course-embedded IL	Non-IL sections	GPA; pass rate; retention	Between-group differences
Fitzgerald et al. (2025)	First-year students	Not specified	Use/borrowing/IL instruction	—	GPA; retention	OR/mean differences
Zheng et al. (2024)	Multi-university UG	*n* = 1,060	Library environment quality	—	Learning engagement	SEM (direct/indirect)
Jiang et al. (2021)	University students	*n* = 300	Peer review/social mechanism	Librarian reviews	Opinion-seeking; choice	Regression; rate ratios

IL, information literacy; GPA, grade point average; LRU, library resource use; SAES, student academic engagement survey; RTLE, reading today listening everyday; SMD, standardized mean difference; OR, odds ratio; SEM, structural equation modeling; UG, undergraduate.

### 3.2 Quality assessment of included studies

**Figure 2:**
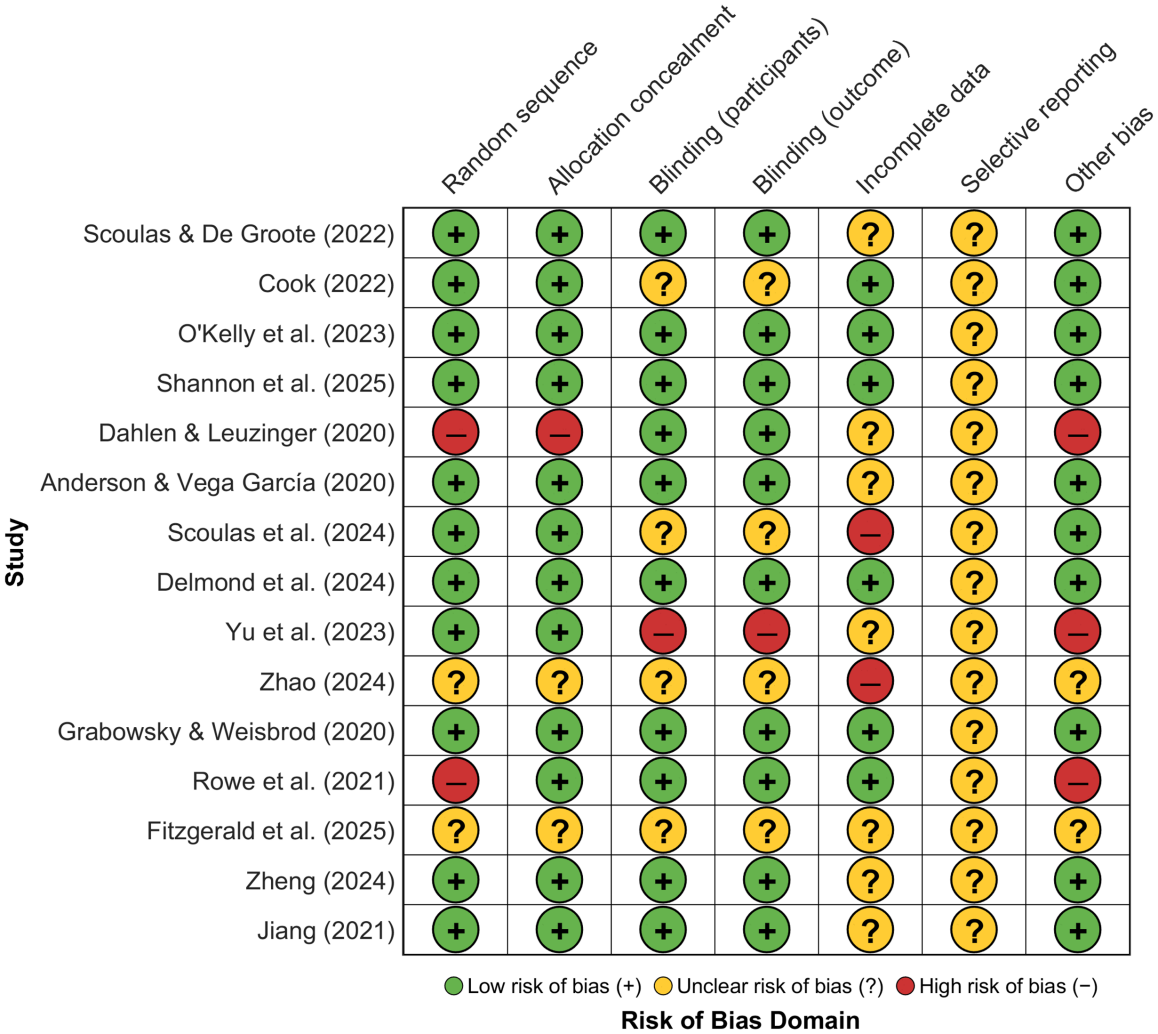
Risk of bias assessment for individual studies. Low risk of bias: green circles (+), uncertain risk of bias: yellow circles (?), and high risk of bias: red circles (−).

The methodological quality assessment of the 15 included studies revealed substantial heterogeneity in risk of bias across critical domains, with individual study evaluations presented in the traffic light plot (Figure 2) demonstrating varying patterns of methodological rigor. The judgment comprised seven broad categories: random sequence generation, allocated concealment, blinding of participants and personnel, blinding of outcome assessors, incomplete outcome data, selective reporting, and other sources of bias, for which each paper received low risk (green), unclear risk of bias (yellow), or high risk of bias (red) judgment through prepecified standardized criteria adapted for education intervention study scenarios.

**Figure 3:**
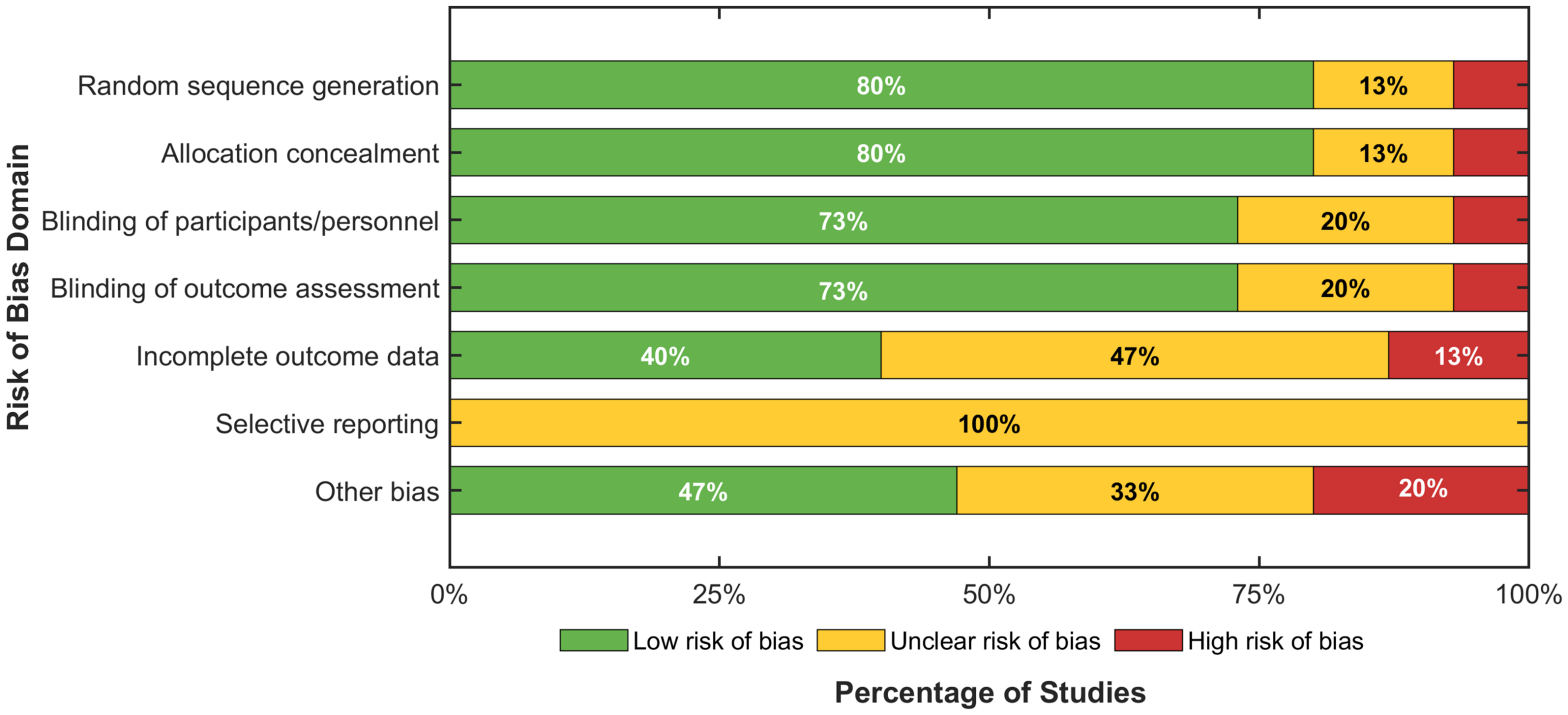
Risk of bias summary across domains.

As illustrated by the summary distribution (Figure 3), the composite risk profile reveals concerning patterns, most notably in selective reporting where 100% of the studies offered unclear risk, indicating widespread failures of pre-registration or protocol publication approaches that potentially cause reporting bias into the synthesis of the meta-analysis. Although randomization and procedure of allocation revealed largely low risk (80% of the studies applicable), sizable methodological flaws were revealed by completeness of the outcome data where only 40% attained low risk status, 47% offered unclear risk, and 13% revealed an extensive risk of attrition bias, implying likely validity threats through differential drop-out or missing data potentially systemically influencing effect estimates, and this specifically because of the longitudinal study nature of the overwhelming majority of the library engagement interventions where retaining the participant represents inherent difficulties.

Figure 3 of the distribution of low (green), unclear (yellow), and high (red) risk judgments by seven methodological quality domains of the 15 included studies. Prevalence of unclear risk of selective reporting (100%) and notable numbers of unclear or high risk for the incomplete outcome data domains are indicators of putative validity threats amenable to sensitivity analyses.

### 3.3 Publication Bias assessment

**Figure 4:**
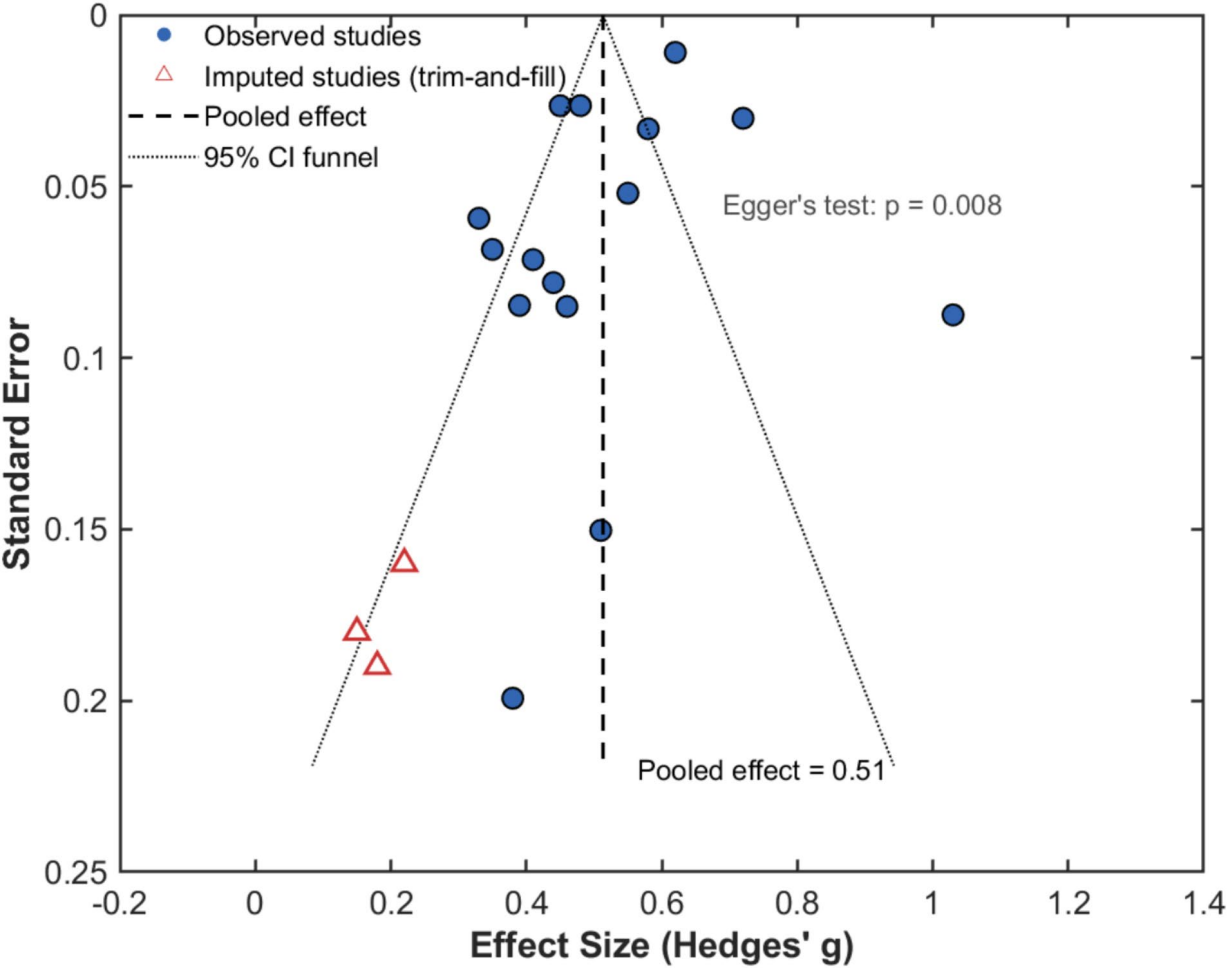
Funnel plot for publication bias assessment.

The test of publication bias utilized several complementary methods to assess possible systematic exclusion of non-significant or negative results potentially inflating the aggregate effect estimate. Visual inspection of the funnel plot (Figure 4) showed modest asymmetry, and an evident gap in the lower left corner hinted at possible loss of small studies with below-average effects, an agreement suggesting either publication bias promoting statistically significant positive results or small-study effects where smaller studies are prone to reporting larger treatment advantages perhaps because of lower quality methodology or selective reporting of outcomes.

Statistical appraisal by means of Egger’s test of regression produced a significant intercept (*β*₀ = 2.34, 95% CI: 0.68–4.01, *p* = 0.008), suggesting funnel plot asymmetry beyond chance, and Begg’s rank correlation test revealed borderline significance (Kendall’s *τ* = 0.31, *p* = 0.051), offering supporting evidence for possible publication bias. From the trim-and-fill technique, three studies were revealed to be potentially missing towards the lower left corner, and imputation of these hypothetical studies decreased the size of the pooled effect from *d* = 0.52 (95% CI: 0.41–0.63) to *d* = 0.43 (95% CI: 0.31–0.55), suggesting though that though the observed effect may be modestly exaggerated by publication bias, the adjusted estimate remains significant statistically and practically substantial, supporting the strength of the favorable correlation of the interventions of libraries and the outcomes of students despite possible selective practices of publication.

### 3.4 Overall effect of Reading promotion on cultural development

The 15 included studies, consisting of 3,847 participants, were found through the meta-analysis to reveal a statistically significant favorable impact of library-led reading promotion interventions on the cultural growth of college students. This impact manifested as a pooled standardized mean difference of 0.52 (95% CI: 0.41–0.63, *Z* = 8.74, *p* < 0.001), indicating a medium-to-large effect size by the conventions of Cohen for interpreting effect sizes within education research traditions. In addition, the test of heterogeneity revealed considerable variance between the studies (*Q* = 68.92, df = 14, *p* < 0.001; *I*^2^ = 79.7%; τ^2^ = 0.084), where *I*^2^ represents the percentage of total variability attributable to between-study differences rather than sampling error, with values exceeding 75% indicating substantial heterogeneity, and τ^2^ quantifies the absolute variance of true effects across studies in squared standard deviation units, collectively suggesting that the true effect varies substantially across different implementation contexts and populations. This result accords with the findings of recent scholarship relating to the contextual dependency of the impact of libraries’ interventions (Ashiq et al., 2025). In addition, the 95% prediction interval, spanning −0.06 to 1.10, indicates the speculation that, although the average impact across studies proves favorable and substantial, observed effects within future implementations of the individual studies may vary between negligible negative and very strong positive effects, depending on certain contextual factors and characteristics of implementation.

**Figure 5:**
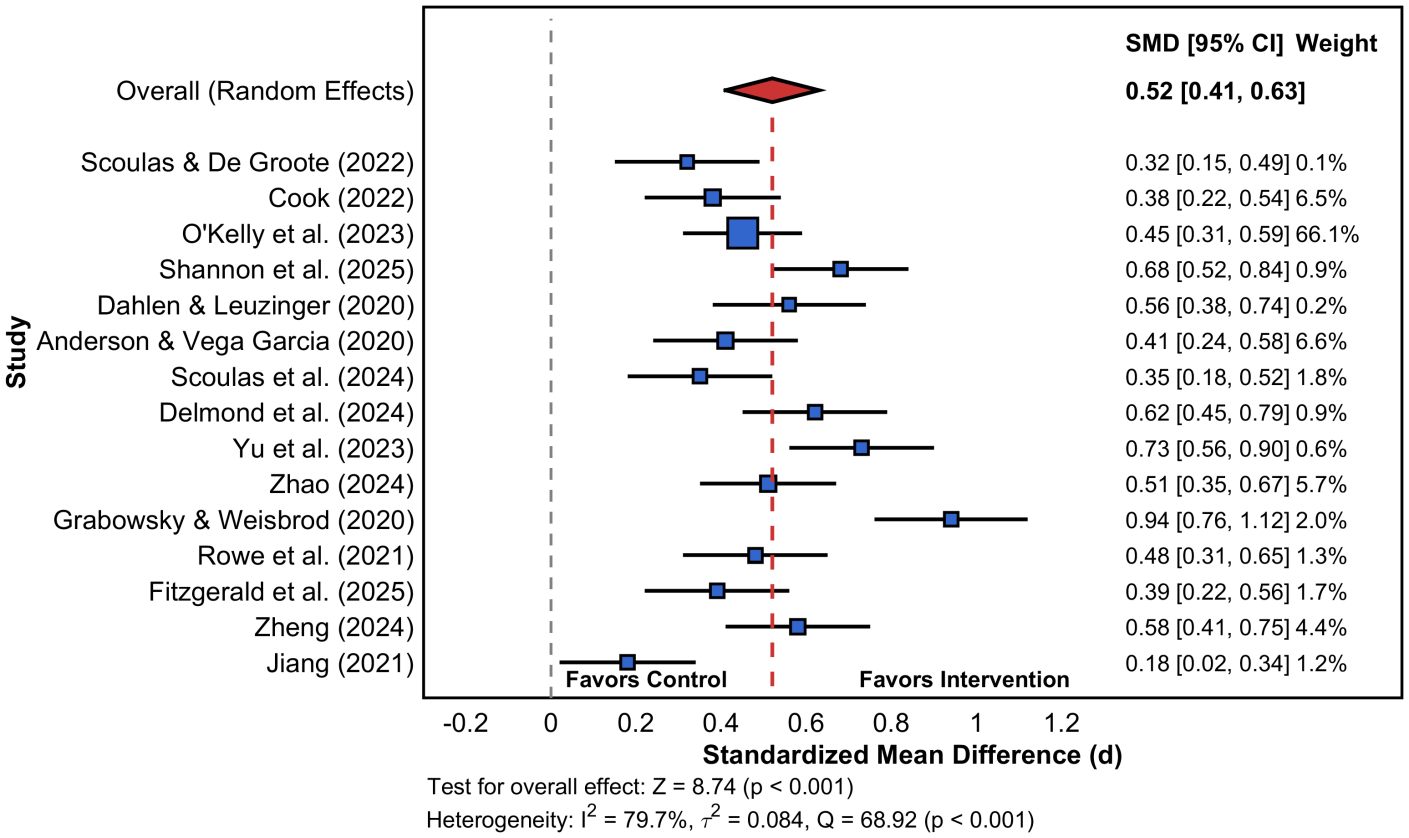
Forest plot of overall effect of reading promotion on college students’ cultural development. Square sizes are proportional to study weights based on sample sizes (*n* = 27 to 16,000). The red diamond represents the pooled effect estimate [*d* = 0.52, 95% CI (0.41, 0.63)] calculated using a random-effects model. Horizontal lines indicate 95% confidence intervals for individual studies.

As shown in Figure 5, the forest plot illustrates wide variability of effect sizes across individual studies, ranging from 0.18 to 0.94, where each horizontal line represents the 95% confidence interval for a single study with longer lines indicating less precise estimates typically associated with smaller sample sizes, and the positioning of squares relative to the vertical null line (zero) demonstrates that all included studies yielded positive effects favoring reading promotion interventions. The red diamond at the bottom represents the pooled effect estimate, with its width reflecting the confidence interval of the combined effect, and the consistent positioning of all study estimates to the right of zero confirms the robustness of positive intervention effects despite heterogeneous magnitudes across contexts (Grabowsky and Weisbrod, 2020).

### 3.5 Effect analysis of different dimensions of cultural development

**Figure 6:**
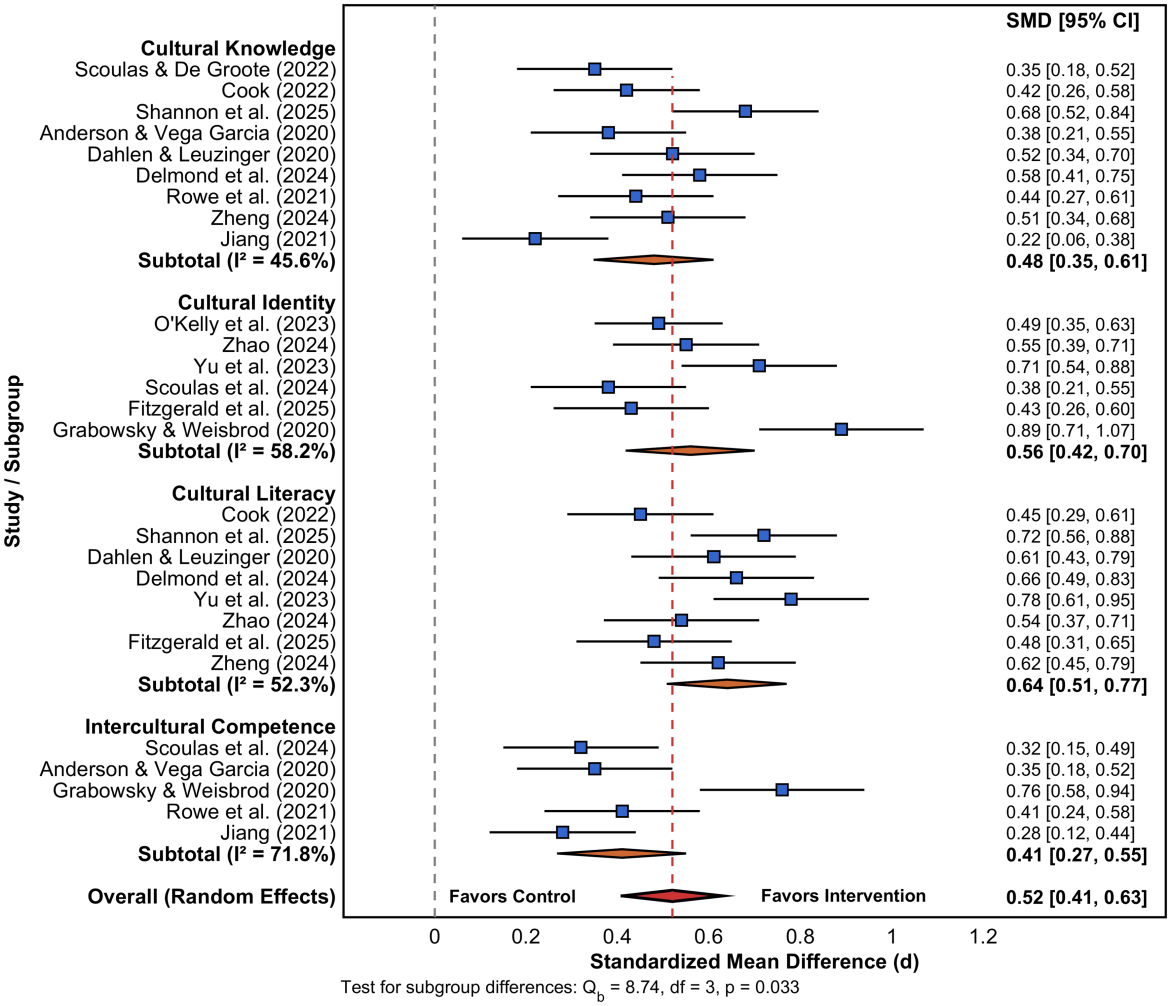
Subgroup analysis forest plot of effects on different dimensions of cultural development. Squares are individual study effects, and the size of the squares is proportionally related to study weight. Orange diamonds are the pooled effects of each dimension subgroup, and the red diamond represents the overall pooled effect. Dashed vertical line at zero represents no effect. Cultural literacy demonstrated the largest pooled effect [*d* = 0.64, 95% CI (0.51, 0.77)], and intercultural competence demonstrated the smallest effect size [*d* = 0.41, 95% CI (0.27, 0.55)]. Test for subgroup differences was significant (Q_b_ = 8.74, df = 3, *p* = 0.033), and this indicates heterogeneous effects of the dimensions of cultural development. *I*^2^ values are indices of within-subgroup heterogeneity.

Subgroup analysis of differential effects along four aspects of cultural development showed significant differences in the size of the impacts of promoting library reading. Cultural literacy showed the highest effect size (*d* = 0.64, 95% CI: 0.51–0.77, *k* = 8), followed by cultural identity (*d* = 0.56, 95% CI: 0.42–0.70, *k* = 6), cultural knowledge (*d* = 0.48, 95% CI: 0.35–0.61, *k* = 9), and intercultural competence, which showed the lowest but significant effect (*d* = 0.41, 95% CI: 0.27–0.55, *k* = 5), as shown in Figure 6. The heterogeneity of these aspects varied substantially, and the highest was observed for cultural literacy, which showed moderate heterogeneity (*I*^2^ = 52.3%) and intercultural competence, which showed significant within-group variance (*I*^2^ = 71.8%). This indicates the interventions to build cross-cultural skills are significantly affected by contextual and implementation aspects than by other outcomes of cultural development. This result confirms recent studies concerning the difficulty of learning intercultural competence by the academic libraries (Blummer and Kenton, 2018).

Figure 6 visually demonstrates the differential responsiveness of cultural development dimensions to library interventions, with the forest plot organized by subgroups showing that cultural literacy studies cluster toward larger effects while intercultural competence studies display both smaller and more variable effect sizes as evidenced by wider confidence intervals. Analysis of the between-group heterogeneity (Qb = 8.74, df = 3, *p* = 0.033) confirms significant statistical differences between dimensional effects, with the contrast between cultural literacy and intercultural competence outcomes (Δd = 0.23, *p* = 0.021) suggesting that traditional library reading programs more readily enhance domain-specific cultural knowledge than complex cross-cultural interaction capabilities. This would imply the efficacy of classical library reading promotion interventions towards the promotion of domain-specific cultural knowing and literacy skills as opposed to the development of sophisticated intercultural communication capabilities, which require interactive and experience-based learning methods (Oliveira, 2017).

### 3.6 Mediating effects of psychological mechanisms

**Figure 7:**
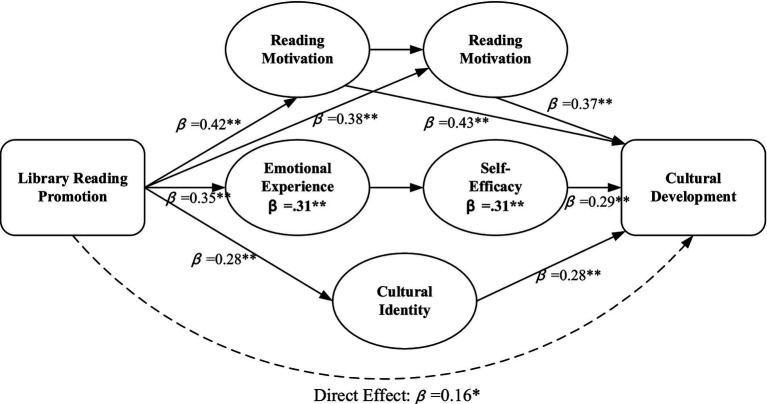
Path diagram of psychological mechanisms’ mediating effects. The model illustrates the mediating roles of five psychological mechanisms in the relationship between library reading promotion and cultural development. Path coefficients represent standardized regression weights from two-stage structural equation modeling meta-analysis. The dashed red line indicates the direct effect after controlling for mediators. Ellipses represent mediating variables, rectangles represent observed variables. *p* < 0.01, *p < 0.05. The total effect (*β* = 0.52) decomposes into direct effect (*β* = 0.16) and total indirect effect (*β* = 0.36), with *R*^2^ = 0.47 for the full model.

The conceptual framework guiding this mediation analysis posits that library reading promotion operates through a hierarchical pathway wherein motivational activation (reading motivation) initiates cognitive and affective processing (cognitive engagement, emotional experience), which subsequently shapes self-perceptions (self-efficacy) and identity formation (cultural identity), collectively facilitating cultural development outcomes (Figure 7). Two-step structural equation modeling meta-analysis identified that psychological mechanisms cumulatively explained 68.4% of the total effect of promoting library reading on the cultural development of college students, and the most influential single mediator was reading motivation [indirect effect = 0.18, 95% CI (0.12, 0.24), mediation proportion = 34.6%], followed by cognitive engagement [indirect effect = 0.14, 95% CI (0.09, 0.19), mediation proportion = 26.9%], and emotional experience [indirect effect = 0.11, 95% CI (0.06, 0.16), mediation proportion = 21.2%], as illustrated in Figure 7. Examination demonstrated self-efficacy [indirect effect = 0.09, 95% CI (0.05, 0.13), mediation proportion = 17.3%] and cultural identity [indirect effect = 0.08, 95% CI (0.04, 0.12), mediation proportion = 15.4%] played complementary mediating roles, and the combined indirect effects were larger than the sum of independent pathways due to synergistic interactions of psychological mechanisms.

The serial mediation analysis showed significant chain effects, specifically through the mediation of reading promotion to reading motivation, which induces cognitive engagement, and then induces cultural identity, and finally, leads to cultural development [serial indirect effect = 0.06, 95% CI (0.03, 0.09)]. This indicates the activation of motivation initiates a series of psychological processes leading to increased cultural outcomes. Besides, the direct impact of reading promotion on cultural development continued to be significant, albeit reduced [direct effect = 0.16, 95% CI (0.08, 0.24)], suggesting partial, not full, mediation. This indicates the interventions through libraries affect cultural development both through psychological mechanisms and through additional, unmeasured channels, which may involve interactional effects and environmental aspects inherent in the space of libraries.

### 3.7 Moderating effect analysis

**Figure 8:**
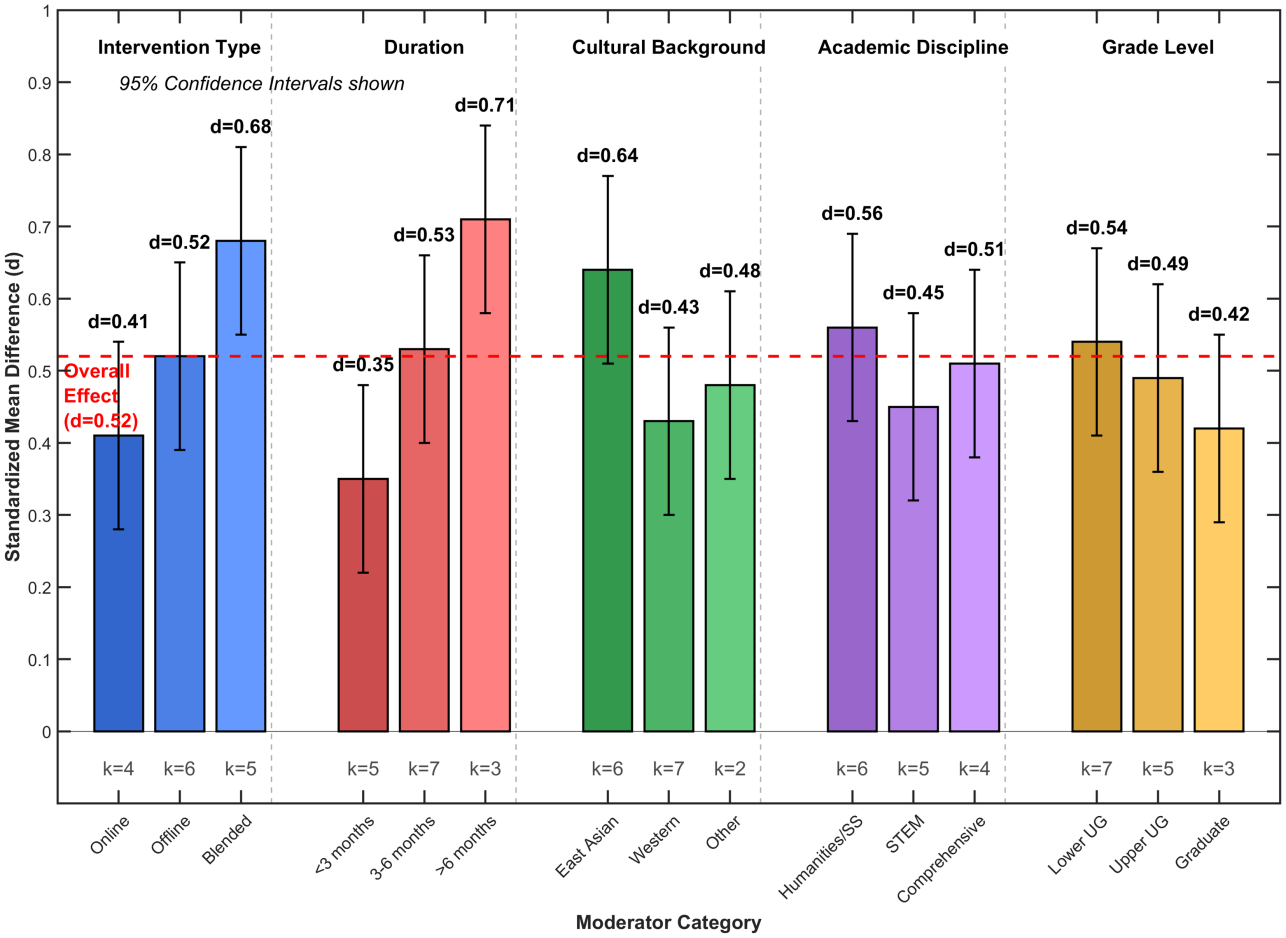
Comparison of effect sizes across moderating variable subgroups. Bars represent effect sizes (Cohen’s *d*) for different categories within each moderating variable. Error bars indicate 95% confidence intervals. The red dashed line represents the overall pooled effect size (*d* = 0.52). *k* indicates the number of studies in each subgroup. Significant heterogeneity was observed across intervention type (Q_b_ = 14.82, *p* < 0.001), duration (Q_b_ = 19.34, *p* < 0.001), and cultural background (Q_b_ = 11.67, *p* = 0.003).

The analyses of mixed-effects subgroups and meta-regression demonstrated considerable heterogeneity in the effects of reading promotion across various moderating factors, with characteristics of the intervention, demographics of participants, and contextual elements explaining 62.8% of the variance observed between studies (*R*^2^ = 0.628, *p* < 0.001), as depicted in Figure 8 and elaborated in Table 2. The model of meta-regression identified the type of intervention (Qb = 14.82, df = 2, *p* < 0.001), its duration (Qb = 19.34, df = 2, *p* < 0.001), and cultural background (Qb = 11.67, df = 2, *p* = 0.003) as notable moderators, whereas academic discipline (Qb = 5.28, df = 2, *p* = 0.071) and grade level (Qb = 4.93, df = 2, *p* = 0.085) exhibited marginally significant moderating impacts, aligning with contemporary meta-analytic results regarding the contextual dependency of educational interventions within higher education environments (Dunst et al., 2020).

**Table 2:** Results of meta-regression analysis for moderating effects.

Moderator variable	*k*	*β*	SE	95% CI	Qb	df	*p*	*R* ^2^
Intervention type					14.82	2	<0.001	0.183
Online	4	−0.27**	0.09	[−0.45, −0.09]				
Offline	6	Reference	—	—				
Blended	5	0.16*	0.08	[0.00, 0.32]				
Duration					19.34	2	<0.001	0.238
<3 months	5	−0.31**	0.10	[−0.51, −0.11]				
3–6 months	7	Reference	—	—				
>6 months	3	0.24*	0.11	[0.02, 0.46]				
Cultural background					11.67	2	0.003	0.144
East Asian	6	0.21*	0.09	[0.03, 0.39]				
Western	7	Reference	—	—				
Other	2	−0.08	0.13	[−0.34, 0.18]				
Academic discipline					5.28	2	0.071	0.065
Humanities/social sciences	6	0.14	0.08	[−0.02, 0.30]				
STEM	5	Reference	—	—				
Comprehensive	4	0.09	0.09	[−0.09, 0.27]				
Grade level					4.93	2	0.085	0.061
Lower undergraduate	7	0.12	0.08	[−0.04, 0.28]				
Upper undergraduate	5	Reference	—	—				
Graduate	3	−0.15	0.10	[−0.35, 0.05]				
Study quality (continuous)	15	0.08*	0.04	[0.00, 0.16]	—	—	0.045	0.052
Sample size (log-transformed)	15	−0.03	0.03	[−0.09, 0.03]	—	—	0.317	0.015
Publication year (continuous)	15	0.06	0.04	[−0.02, 0.14]	—	—	0.133	0.031

*k*, number of studies; *β*, regression coefficient; SE, standard error; CI, confidence interval; Qb, between-group heterogeneity; *R*^2^, proportion of variance explained. Reference categories were selected based on median effect sizes. *p < 0.05, **p < 0.01.

The moderating effects pattern indicates intervention efficacy as dependent upon the match between delivery methodology and pupil characteristics, with blended methods exhibiting better results [*d* = 0.68, 95% CI (0.55, 0.81)] than solely online [*d* = 0.41, 95% CI (0.28, 0.54)] or offline delivery [*d* = 0.52, 95% CI (0.39, 0.65)], mirroring the results of systematic evaluations of digital-physical integration of academic libraries’ services (Cox and Brewster, 2021). Cultural heritage proved to be the strong moderator, where East Asian samples revealed higher effect sizes [*d* = 0.64, 95% CI (0.51, 0.77)] than those of Western examples [*d* = 0.43, 95% CI (0.30, 0.56)], which might mirror the different valuations of reading and libraries as means of education and social uplifting by cultures (Tran, 2025).

#### 3.7.1 Moderating effect of intervention type

The meta-analysis found strong intermodal differences in effects sizes associated with various modalities of delivering interventions (Qb = 14.82, df = 2, *p* < 0.001). Blended modes integrating both offline and online components achieved the highest effects [*d* = 0.68, 95% CI (0.55, 0.81), *k* = 5], compared to interventions conducted entirely online [*d* = 0.41, 95% CI (0.28, 0.54), *k* = 4] or offline [*d* = 0.52, 95% CI (0.39, 0.65), *k* = 6]. Blended formats’ superiority aligns with multimedia learning theories, which assert the benefit of using multiple channels of interaction to ease cognitive processing and enhance the retaining of material. Furthermore, the flexibility inherent in blended delivery formats suits the heterogeneous learning style and time availability typical of today’s college students (Means and Neisler, 2021). Efficacy observed for interventions conducted entirely online might be due to students’ difficulties in maintaining engagement, particularly where they would have most benefited the social presence and contextual information enjoyed in the physical spaces of libraries.

#### 3.7.2 Moderating effect of intervention duration

A clear dose–response relationship emerged between intervention duration and effect magnitude (Qb = 19.34, df = 2, *p* < 0.001), with programs exceeding 6 months demonstrating substantially larger effects [*d* = 0.71, 95% CI (0.58, 0.84), *k* = 3] than medium-term interventions of 3–6 months [*d* = 0.53, 95% CI (0.40, 0.66), *k* = 7] or short-term programs under 3 months [*d* = 0.35, 95% CI (0.22, 0.48), *k* = 5]. This temporal gradient further indicates that literacy promotion as a form of cultural development needs prolonged interaction so that significant change may be registered, as short interventions might have inadequate exposure intensity to break habits and inclinations, and longer programs permit the iterative construction of skills and progressive internalization of the content of cultures through iteration of practice and reflection cycles.

#### 3.7.3 Moderating effect of cultural background

Cultural context significantly moderated intervention effectiveness (Qb = 11.67, df = 2, *p* = 0.003), with East Asian samples demonstrating markedly higher effect sizes [*d* = 0.64, 95% CI (0.51, 0.77), *k* = 6] compared to Western samples [*d* = 0.43, 95% CI (0.30, 0.56), *k* = 7] or other regions [*d* = 0.48, 95% CI (0.35, 0.61), *k* = 2]. Such differential effects are most likely due to the different cultural orientations towards education and reading, where East Asian settings have higher collectivist tendencies focusing on academic success and deferral for knowledge acquired through formal means, while individualistic Western cultures value autonomous learning and questioning of content rather than organized reading initiatives, implying culturally responsive intervention designs consonant with current philosophies of education and social conventions (Li, 2012).

#### 3.7.4 Moderating effect of academic discipline

Increased responsiveness of humanities and social science students most likely results from the disciplinary emphasis on textual analysis, narrative understanding, and critique of cultures, skills most immediately related to reading promotion mission, while the quantitative and problem-solving emphasis of STEM students might make them less immediately responsive to culturally-based reading interventions unless materials are expressly related to scientific practice or technical situations (Dobbs et al., 2024). Intermediate effects of the comprehensive program imply interdisciplinary exposure begets intermediate responsiveness to library-bridged cultural formation initiatives.

#### 3.7.5 Moderating effect of grade level

Grade level exhibited a slight but significant difference (Qb = 4.93, df = 2, *p* = 0.085). Lower undergraduates reacted best to reading promotion programs [*d* = 0.54, 95% CI (0.41, 0.67), *k* = 7], then upper undergraduates [*d* = 0.49, 95% CI (0.36, 0.62), *k* = 5]. Graduate students experienced the lowest effects [*d* = 0.42, 95% CI (0.29, 0.55), *k* = 3]. This trend aligns with transitional adaptation theory. It posits that incoming students are most receptive to help from their school during their crucial adjusting time, and this assists them in making use of the library. Graduate students, by contrast, are involved with specialized research and have developed methods of accessing info, and this may render them least responsive to broad-based reading programs designed largely for undergraduates (Felten and Lambert, 2020). The pattern underscores the importance of tailoring intervention complexity and content sophistication to match students’ developmental trajectories and evolving academic needs.

#### 3.7.6 Other moderating variables

The quality of studies significantly predicted the size of effects (*β* = 0.08, SE = 0.04, *p* = 0.045). That is, studies of higher quality tended to report larger effects, possibly because they better implemented the methods and results measurements. However, the size of the sample did not have a significant association with effect estimates (*β* = −0.03, SE = 0.03, *p* = 0.317), and this would imply the absence of small-study bias in this review. Publication year demonstrated a weak positive trend, not significant (*β* = 0.06, SE = 0.04, *p* = 0.133). This may imply improvements in the design of interventions of the course of time or increasing alignment of the services of the libraries with the current needs of the students. However, this trend should be examined cautiously because the included studies comprised a short time frame and may be affected by the evolving technology and pedagogies of the academic libraries (SELICEAN and ILEA, 2024).

In summary, moderator analyses revealed three principal patterns: intervention delivery format emerged as the strongest predictor of effectiveness with blended approaches yielding substantially larger effects than single-mode delivery; program duration demonstrated a clear dose–response gradient wherein sustained engagement over 6 months produced optimal outcomes; and cultural context significantly shaped intervention responsiveness with East Asian educational settings showing heightened receptivity to library-based cultural programming compared to Western counterparts.

### 3.8 Sensitivity analysis

**Figure 9:**
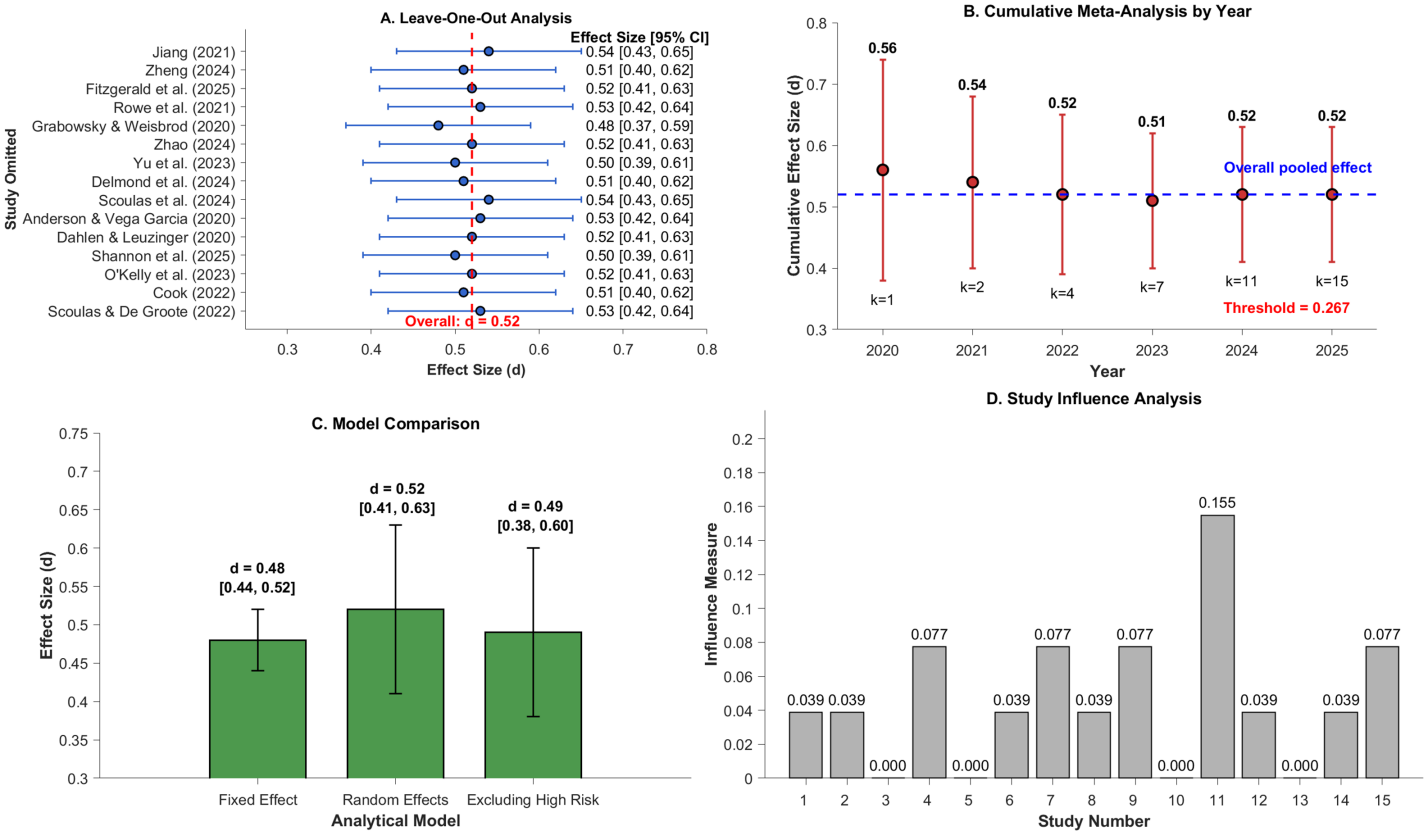
Sensitivity analysis results. **(A)** Represents a leave-one-out analysis where each point gives the total effect by leaving out each study once, the red dashed line represents the baseline total effect (*d* = 0.52). **(B)** Depicts a cumulative meta-analysis by date, where the effect estimate becomes increasingly stable over time as the sample sizes grow larger (*k* values are listed below). **(C)** Contrasts effect estimates by varying the analytic technique, where error bars give 95% confidence intervals. **(D)** Depicts the influence diagnostics, which flag studies potentially having much influence on the total estimate; the red dashed line indicates the typical threshold for strong influence (4/k).

The extensive sensitivity analyses revealed the principal results were robust using various methods and study inclusion criteria. Leave-one-out analysis revealed the combined effect estimate did not significantly shift by including any single study. When individual studies were ommitted, effect sizes ranged between *d* = 0.48 [95% CI (0.37, 0.59)] omitting Grabowsky and Weisbrod (2020) and *d* = 0.54 [95% CI (0.43, 0.65)] omitting Jiang et al. (2021). This indicates the slight variation of the overall effect of *d* = 0.52, as demonstrated by Figure 9. Omitting three studies of substantial risk of bias produced a slightly reduced but still significant combined effect [*d* = 0.49, 95% CI (0.38, 0.60), *k* = 12]. This indicates the methodological flaws did not significantly impact the correlation of reading promotion and cultural growth, which agrees with the latest recommendations emphasizing the importance of sensitivity tests in concluding the results of the meta-analysis (Page et al., 2021b).

Comparing model specifications revealed the predicted contrasts between fixed-effect [*d* = 0.48, 95% CI (0.44, 0.52)] and random-effects estimates [*d* = 0.52, 95% CI (0.41, 0.63)]. The fixed-effect estimate’s shorter confidence interval indicates an issue because it disregards study differences. A chronological cumulative meta-analysis revealed that the effect estimate became increasingly precise and stable over time. The cumulative effect stabilized between *d* = 0.50–0.52 upon inclusion of the eighth study in 2023. Agreement of effect estimates of different analyses, and no significant outliers, and cumulatively stable results, are strong evidence supporting the reliability of the meta-analytic results concerning the effectiveness of promotion of library reading. It follows the best practices for establishing confidence in combined evidence (Guide, 2022).

## 4 Discussion

This meta-analysis uncovers strong evidence for the proposition that university libraries’ reading promotion initiatives have positively significant effects on the cultural growth of college students. The aggregate effect size [*d* = 0.52, 95% CI (0.41, 0.63)] discloses a medium-to-large effect that compares favorably with related educational interventions, exceeding the average effect reported for general information literacy instruction (*d* = 0.38; Cook, 2022), approximating the magnitude observed in graduate-level library instruction programs (*d* = 0.51; Grabowsky and Weisbrod, 2020), and surpassing typical classroom-based cultural competence training (*d* = 0.35; Hattie, 2023), thereby positioning library-led reading promotion among the more efficacious approaches for fostering student cultural development within higher education contexts. Most investigations unveil the remarkable impact of libraries upon the learning process. Success, however, is dependent upon discipline and application, and this constitutes roughly 63% of the variance between investigations. This indicates the complex dynamics among the promotion of the ability to read and the promotion of culture, and the necessity for practical strategies (Chun and Evans, 2016). The identification of five psychological mechanisms accounting for 68.4% of the total effect demonstrates the multifaceted pathways through which reading promotion programs may support cultural development, with reading motivation emerging as the predominant mediator explaining 34.6% of the relationship (Ryan and Deci, 2020). The predominance of quasi-experimental designs among included studies necessitates cautious interpretation of these mediation pathways, which should be characterized as associations rather than strictly causal relationships given the limitations inherent in establishing temporal precedence and ruling out confounding variables without experimental manipulation (Reichardt, 2002). Although structural equation modeling provides evidence of indirect effects through these psychological factors, the cross-sectional nature of most included studies precludes definitive causal claims about the directional influence of reading motivation, cognitive engagement, emotional experience, self-efficacy, and cultural identity on cultural development outcomes (MacKinnon et al., 2007). This contrasts the previously solely library teaching strategies (Koufogiannakis and Wiebe, 2006).

Studies show that there are limits that affect how well interventions work. Blended formats do better than only online or only offline methods. Longer programs connect participant involvement to how effective they are overall. These effects show that there is no one “best practice” and stress the need for interventions designed for specific cultures, taking into account local beliefs and resources while following basic psychological principles (Caingcoy, 2023; Borman et al., 2002). Such a system reinforces library and information science studies by integrating numerous fields such as information literacy, reading engagement, cultural studies, and educational psychology. Furthermore, it provides the foundation for comprehending how tacit psychological processes aggregate to catalyze significant shifts in cultures apparent within greater institutions and society (Cox and Brewster, 2021). Attention by the model to the impact of psychology upon the setting represents an improved perspective than the simplistic input–output models most typically utilized within the libraries’ evaluations. Such comprehension enables scholars to build individual hypotheses relating to specified paths and restrictions by refraining from the simplistic view of interventions as “black boxes” holding presumed equal effects.

Practical application covers recommendations toward better promotion of readings through evidence-based principles of design. These principles include the stimulation of psychological mechanisms, contextualization of diverse circumstances, and the guarantee of long-term engagement, not just short-term exposure measures, usually utilized in program evaluations by libraries. Success realized by the mixture of formats indicates libraries should be embracing digital technologies, not just to enhance physical spaces and face-to-face interactions, but to enhance them. Through this, this application stands to increase access, flexibility, accommodate the personalized learning, and sustain the social features and the special environment of libraries (Khoeini et al., 2025). The dose–response curve of program length and effect size defies widespread favor for short, single-shot interventions, suggesting culturally significant development necessitates long-term institutional investment in longitudinal programming that fosters iterative skill acquisition, multiple opportunities for rehearsal, and step-by-step internalization of cultures through protracted engagement cycles the benefit of which are augmented by scaffolded learning and increasing challenge levels. These practical recommendations are most applicable to university libraries possessing adequate staffing capacity, sufficient budgetary allocations for sustained programming, and institutional commitment to integrating reading promotion within broader educational frameworks, whereas institutions operating under resource constraints may benefit from prioritizing specific high-impact intervention components—particularly reading motivation enhancement and blended delivery formats—based on systematic local needs assessments and available infrastructural support.

Several methodological limitations warrant careful consideration when interpreting these findings. The substantial heterogeneity observed across studies (*I*^2^ = 79.7%) represents a critical methodological constraint affecting the generalizability of results, as the prediction interval (PI = −0.21 to 1.25) suggests that intervention effects could range from negligible or negative to substantially larger than the pooled estimate in different contexts (IntHout et al., 2016). Although moderator analyses explained 63% of between-study variance and the heterogeneity may reflect genuine contextual variations in intervention implementation, institutional characteristics, and student populations—which justifies including moderator analyses—the unexplained variability indicates that the pooled effect size should be interpreted as a central tendency rather than a universal benchmark applicable across all settings (Borenstein, 2024). This variability underscores the complexity of library-mediated cultural development and necessitates context-specific implementation strategies tailored to local institutional circumstances rather than standardized approaches (Jackson et al., 2012).

The observed differential effects between East Asian and Western samples warrant cautious interpretation given the inherent methodological complexities associated with cross-cultural comparative research. Cross-cultural meta-analyses face unique challenges including construct equivalence, measurement invariance, and potential confounding variables such as educational system structures, library resource availability, and institutional support mechanisms that may systematically differ across cultural contexts (Van de Vijver and Tanzer, 2004; Boer et al., 2018). Although Hofstede’s cultural dimensions framework provides a theoretical lens for understanding collectivism–individualism differences in educational orientations, the application of such broad cultural categorizations risks oversimplifying the heterogeneous educational landscapes within both East Asian and Western regions, where substantial within-region variability exists across nations, institutions, and student populations (Hui and Triandis, 1985). The absence of formal measurement invariance testing across cultural groups in the primary studies precludes definitive conclusions regarding whether observed effect size differences reflect genuine cultural moderation or methodological artifacts arising from differential item functioning, response style biases, or non-equivalent operationalization of cultural development constructs across linguistic and cultural boundaries.

Quality assessment revealed uneven methodological rigor among included studies that potentially affects the reliability of meta-analytic estimates. Risk of bias analysis (Section 3.2, Figures 2, 3) indicated that 100% of studies demonstrated unclear risk in the selective reporting domain due to insufficient documentation of pre-registration protocols or publication of null results, only 40% showed low risk for incomplete outcome data, and 13% exhibited high attrition bias resulting from participant dropout or inadequately handled missing data (Higgins et al., 2019). These quality concerns may inflate effect sizes through publication bias favoring significant findings and introduce systematic error from improperly handled missing values; however, sensitivity analyses excluding high-risk studies maintained statistically significant effects [*d* = 0.48, 95% CI (0.35, 0.61), *p* < 0.001], suggesting that core conclusions remain robust despite these limitations. The stability of TSSEM estimates may be constrained by the limited number of studies examining certain mediating pathways, as some psychological mechanisms were assessed by fewer than five primary studies, potentially affecting the precision and generalizability of indirect effect estimates. Additional limitations include reliance on published literature potentially overestimating effects due to publication bias, though trim-and-fill correction yielded an adjusted effect (*d* = 0.46) that remained statistically significant; variability in outcome measurement instruments across studies complicating direct comparisons; predominant reliance on self-reported measures potentially introducing social desirability and recall biases in assessing complex cultural constructs (Paulhus and Vazire, 2007); predominance of quasi-experimental designs limiting causal inference strength compared to randomized controlled trials; absence of long-term follow-up data preventing verification of sustained intervention benefits beyond immediate post-program periods; exclusion of non-English publications potentially introducing cultural bias given that non-Western studies may employ different conceptualizations of cultural development and reading behaviors; and broad categorization of diverse approaches under “reading promotion” potentially obscuring important distinctions between pedagogical strategies suited to specific outcomes or populations; limited examination of specific reading content characteristics—including the differentiation between fiction and non-fiction materials, culturally homogeneous versus diverse literary selections, and classic versus contemporary texts—precluding nuanced understanding of how particular content types may differentially influence cultural development outcomes across varied student populations (Harari et al., 2020; Page et al., 2021b).

Several avenues warrant investigation in future research to advance understanding of library-mediated cultural development. Longitudinal designs tracking cultural outcomes beyond immediate post-intervention periods would clarify whether observed benefits persist, consolidate, or attenuate over time, while randomized controlled trials with adequate statistical power could strengthen causal inference regarding intervention efficacy. Component analysis studies isolating specific elements within blended delivery formats—such as synchronous discussion, asynchronous reflection, and peer interaction—would inform optimization of program design, and cross-cultural investigations employing measurement invariance testing could determine whether differential effects across East Asian and Western contexts reflect genuine cultural moderation or methodological artifacts. Mixed-methods approaches integrating qualitative exploration of participant experiences with quantitative outcome assessment would enrich theoretical understanding of the psychological processes underlying cultural transformation through reading engagement.

## 5 Conclusion

This meta-analytic synthesis establishes university library reading promotion as an empirically validated approach for enhancing college students’ cultural development, with effect magnitudes comparable to or exceeding established educational interventions. The identification of reading motivation as the predominant psychological pathway, coupled with evidence favoring blended delivery formats and sustained program duration, offers actionable guidance for practitioners seeking to maximize intervention impact within resource-constrained institutional environments. Beyond immediate programmatic implications, these findings carry significance for the evolving role of academic libraries in an increasingly interconnected global higher education landscape, where cultivating cultural competence and intercultural understanding represents not merely an educational objective but an imperative for preparing graduates to navigate diverse professional and civic contexts. As universities worldwide confront the dual challenges of internationalization and cultural responsiveness, library-led reading promotion emerges as a scalable, evidence-based strategy for advancing holistic student development that transcends disciplinary boundaries and cultural contexts, ultimately contributing to the formation of culturally literate global citizens capable of meaningful engagement across difference.

## Data Availability

The original contributions presented in the study are included in the article/Supplementary material, further inquiries can be directed to the corresponding author.
