# Supplementary material for: University library reading promotion and college students’ cultural development: a meta-analysis of psychological mechanisms and moderating effects
Source: Front Psychol. 2026 Mar 19;17:1730597. doi: 10.3389/fpsyg.2026.1730597 (PMC13044778; doi:10.3389/fpsyg.2026.1730597)
Supplement: Supplementary file 1 [file Table_1.docx]

# Supplementary Table S1. Coding Protocol and Decision Rules

**A. Study Characteristics**

| **Variable** | **Categories** | **Coding Rule** | **Example** |
| --- | --- | --- | --- |
| Publication Year | Continuous (2020-2025) | Year of publication as reported | O'Kelly et al. (2023) → 2023 |
| Geographic Region | East Asian / Western / Other | East Asian = China, Japan, Korea, Taiwan, Hong Kong, Singapore; Western = USA, UK, Canada, Australia, Western Europe | Zhao (2024): China → East Asian |
| Study Design | Experimental / Quasi-experimental / Observational | Experimental = random assignment; Quasi-experimental = comparison without randomization | Cook (2022) → Quasi-experimental |
| Sample Size | Continuous | Total participants in intervention and control conditions | Scoulas & De Groote (2022): n = 27 |

**B. Intervention Characteristics**

| **Variable** | **Categories** | **Coding Rule** | **Example** |
| --- | --- | --- | --- |
| Intervention Type | Online / Offline / Blended | Online = entirely digital; Offline = entirely physical; Blended = both online and in-person components | Shannon et al. (2025): Asynchronous workshop → Online |
| Intervention Duration | <3 months / 3-6 months / >6 months | <3 months = ≤12 weeks; 3-6 months = 13-26 weeks; >6 months = >26 weeks | Delmond et al. (2024): single module → <3 months |
| Intervention Content | IL / Bibliotherapy / Reading Circles / Culturally Responsive / Mixed | Coded based on primary program description | Cook (2022): one-shot IL → Information Literacy |

**C. Participant Characteristics**

| **Variable** | **Categories** | **Coding Rule** | **Example** |
| --- | --- | --- | --- |
| Academic Discipline | Humanities & Social Sciences / STEM / Comprehensive | Based on reported participant major; Comprehensive = mixed sample | Zheng et al. (2024) → Comprehensive |
| Grade Level | Lower UG / Upper UG / Graduate | Lower UG = Year 1-2; Upper UG = Year 3-4; Graduate = Master's/Doctoral | Grabowsky & Weisbrod (2020) → Graduate |

**D. Outcome Variables**

| **Variable** | **Categories** | **Coding Rule** | **Example** |
| --- | --- | --- | --- |
| Cultural Development Dimension | Cultural Knowledge / Identity / Literacy / Intercultural Competence | Knowledge = factual understanding; Identity = self-perception; Literacy = interpretive skills; Competence = cross-cultural abilities | Zhao (2024) → Cultural Literacy |
| Measurement Type | Standardized / Researcher-developed / Academic Proxy | Standardized = validated instrument; Academic Proxy = GPA or grades | Anderson & Vega García (2020): GPA → Academic Proxy |

**E. Psychological Mechanisms**

| **Variable** | **Operational Definition** | **Measurement Indicators** |
| --- | --- | --- |
| Reading Motivation | Intrinsic and extrinsic drives to engage with reading materials | Self-reported interest scales, reading frequency, voluntary reading time |
| Cognitive Engagement | Active mental processing during reading activities | Deep processing strategies, critical thinking measures |
| Emotional Experience | Affective responses elicited through reading | Reading enjoyment, emotional resonance scales |
| Self-Efficacy | Confidence in ability to engage with cultural content | Reading self-efficacy scales, academic confidence measures |
| Cultural Identity | Sense of belonging to cultural groups through reading | Cultural identification scales, heritage connection measures |

**F. Effect Size Calculation**

| **Source Statistic** | **Conversion Formula** | **Notes** |
| --- | --- | --- |
| Means and SDs | d = (M₁ - M₂) / SD_pooled | SD_pooled = √[(SD₁² + SD₂²) / 2] |
| Correlation coefficient (r) | d = 2r / √(1 - r²) | Used when only correlations reported |
| t-value | d = 2t / √(df) | df = degrees of freedom |
| F-value (two groups) | d = √(F × (n₁ + n₂) / (n₁ × n₂)) | For between-group comparisons |
| Odds Ratio (OR) | d = ln(OR) × √3 / π | For dichotomous outcomes |
| Hedges' g correction | g = d × (1 - 3 / (4 × df - 1)) | Applied to correct small sample bias |

**G. Quality Assessment Coding (RoB 2 / ROBINS-I)**

| **Domain** | **Low Risk** | **Unclear Risk** | **High Risk** |
| --- | --- | --- | --- |
| Random Sequence Generation | Adequate method described | Method not described | Non-random method |
| Allocation Concealment | Allocation concealed | Not described | Allocation predictable |
| Incomplete Outcome Data | <10% attrition, balanced | 10-20% attrition | >20% or differential dropout |
| Selective Reporting | Protocol available, all outcomes reported | No protocol available | Discrepancies identified |

**H. Inter-rater Reliability**

| **Coding Stage** | **Agreement Measure** | **Threshold** | **Resolution Procedure** |
| --- | --- | --- | --- |
| Title/Abstract Screening | Cohen's κ | κ > 0.75 | Consensus discussion |
| Full-text Assessment | Cohen's κ | κ > 0.75 | Third reviewer consultation |
| Data Extraction | Percent Agreement | >90% | Re-extraction and verification |
| Quality Assessment | Cohen's κ | κ > 0.80 | Joint re-assessment |

**Notes:** All coding decisions were made independently by two reviewers. Discrepancies were resolved through consensus discussion. When consensus could not be reached, a third reviewer with expertise in library science education was consulted.
